# Protocol: transient expression system for functional genomics in the tropical tree *Theobroma cacao* L.

**DOI:** 10.1186/s13007-016-0119-5

**Published:** 2016-03-11

**Authors:** Andrew S. Fister, Zi Shi, Yufan Zhang, Emily E. Helliwell, Siela N. Maximova, Mark J. Guiltinan

**Affiliations:** The Huck Institutes of the Life Sciences, The Pennsylvania State University, 422 Life Sciences Building, University Park, PA 16802 USA; Center for Applied Genetic Technologies, University of Georgia, Athens, GA 30602 USA; Department of Electrical Engineering, Princeton University, Princeton, NJ 08544 USA; Department of Botany and Plant Pathology, Center for Genome Research and Biocomputing, Oregon State University, Corvallis, OR 97331 USA; The Department of Plant Science, The Pennsylvania State University, University Park, PA 16802 USA

## Abstract

**Background:**

*Theobroma cacao* L., the source of cocoa, is a crop of significant economic value around the world. To facilitate the study of gene function in cacao we have developed a rapid *Agrobacterium*-mediated transient genetic transformation protocol. Here we present a detailed methodology for our transformation assay, as well as an assay for inoculation of cacao leaves with pathogens.

**Results:**

*Agrobacteriumtumefaciens* cultures are induced then vacuum-infiltrated into cacao leaves. Transformation success can be gauged 48 h after infiltration by observation of green fluorescent protein and by qRT-PCR. We clarify the characteristics of cacao leaf stages and demonstrate that our strategy efficiently transforms leaves of developmental stage C. The transformation protocol has high efficacy in stage C leaves of four of eight tested genotypes. We also present the functional analysis of cacao chitinase overexpression using the transient transformation system, which resulted in decreased pathogen biomass and lesion size after infection with *Phytophthora tropicalis*.

**Conclusions:**

Leaves expressing transgenes of interest can be used in subsequent functional genetic assays such as pathogen bioassay, metabolic analysis, gene expression analysis etc. This transformation protocol can be carried out in 1 day, and the transgenes expressing leaf tissue can be maintained in petri dishes for 5–7 days, allowing sufficient time for performance of additional downstream gene functional analysis. Application of these methods greatly increases the rapidity with which candidate genes with roles in defense can be tested.

**Electronic supplementary material:**

The online version of this article (doi:10.1186/s13007-016-0119-5) contains supplementary material, which is available to authorized users.

## Background

*Theobroma cacao* L., the source of cocoa, is a tree crop of great international economic importance and the center of the multi-billion-dollar chocolate industry. While the tree is native to the Amazon basin [1], approximately 70 % of cocoa is now produced in West Africa, with the remainder coming from South America and Southeast Asia [2, 3]. Each year the crop suffers significant losses to a variety of fungal, oomycete, and viral diseases [4], resulting in significant financial loss for cacao farmers and nations exporting cocoa. Cacao research has benefited from the recent publication of the genome sequences of two genotypes [5, 6]. Availability of this data increases the speed with which putatively important cacao genes can be functionally characterized, which could lead to crop improvement through application of novel breeding strategies or biotechnological approaches [7], although progress with long-generation crops is inherently slow. Accordingly, development of strategies enabling gene characterization is important to expedite the process of genetic improvement of cacao.

*Agrobacterium*-mediated transient and stable plant transformation techniques were developed to enable the introduction of recombinant DNA into plant cells in plants [8, 9]. Whereas transient expression is largely the result of transcription and translation of non-integrated T-DNA, stable transformation by definition implies the integration of T-DNA into the host genome [10]. Transiently transfected plants typically show a peak in expression 2–4 days after infection with *Agrobacterium* which subsequently declines [10], while stable transformation is typically achieved through selection and culturing of transformed tissue, and leads to persistent expression of transgenes [11]. If germ line cells are transformed, integration of T-DNA is heritable [12]. While stable transformation is essential for applications in crop improvement, transient transformation enables rapid testing of gene function, and is therefore an invaluable tool for plant genetics research. Both transformation strategies have been applied to a number of tree crops including cacao [13–20], and it has been applied to enhancement the of disease resistance, abiotic stress response, improvement of quality traits, and general study of functional genetics [21].

Traditional breeding strategies for tree crops are laborious and expensive. For cacao, generation of new varieties through breeding programs can take 15–20 years [3]. A strategy for generation of stable transgenic cacao trees was previously published [16], however even this process takes several years to produce a mature tree that could be used to assay experimentally the effect of a transgene’s overexpression or knockdown. The transient transformation protocol and subsequent functional analysis described here can be performed in a week, and has been used to demonstrate effect of overexpression [13, 20] and knockdown [22] of cacao genes with roles in defense, expression of non-native phosphatidylinositol 3-phosphate binding proteins in cacao [14], and the function of a transcription factor controlling embryogenesis [19].

Here we present the protocol for *Agrobacterium*-mediated transiently transform of detached leaf tissue of *Theobroma cacao*. Growth conditions described here were extensively tested to optimize transformation efficiency. The strategy enables functional gene characterization to be performed in a matter of weeks, rather than the years that would be required to generate a stably transgenic cacao tree.

## Experimental design

**Fig. 1:**
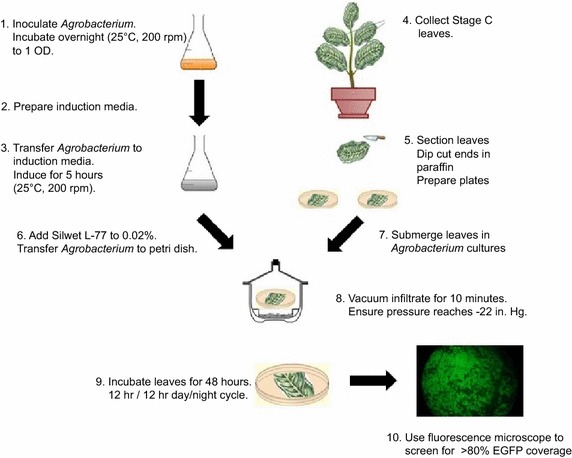
Workflow diagram for transient transformation of cacao leaf tissue

The protocol described here has been used to rapidly screen vectors to measure the effect of gene overexpression or knockdown in cacao leaf tissue [13, 14, 19, 20, 22]. Prior to transformation, binary vector constructs were transferred into competent *Agrobacterium* of strain AGL1 as previously described [23]. Typically the experiment is performed using two vectors: an experimental construct and a control construct (typically pGH00.0126, GenBank: KF018690). Leaves are divided into two sections, one closer to the tip and one closer to the base, such that each leaf can be transformed with both constructs. Preliminary experiments have showed that transformation success usually does not differ significantly between the two sections of a given leaf (data not shown). The two sections of a leaf are simultaneously infiltrated by submerging leaf discs in cultures of *Agrobacterium* and applying a vacuum. Transformation success is evaluated 48 h after infiltration by observing EGFP fluorescence. A leaf is only used for subsequent functional characterization of EGFP is uniformly present across >80 % of the surface area of the control and experimental sections of a given leaf. A workflow diagram of the transient transformation process is depicted in Fig. 1. It is important to note that efficiency of transformation varies significantly between leaves, and proper appraisal of leaf stage is critical for a successful experiment. At least 3 replicates per transgene are typically used for statistical power. In order to ensure that 3–5 leaf sections per construct are successfully transformed, we recommend infiltrating 8–10, anticipating several leaves will not pass the EGFP coverage threshold.

**Fig. 2:**
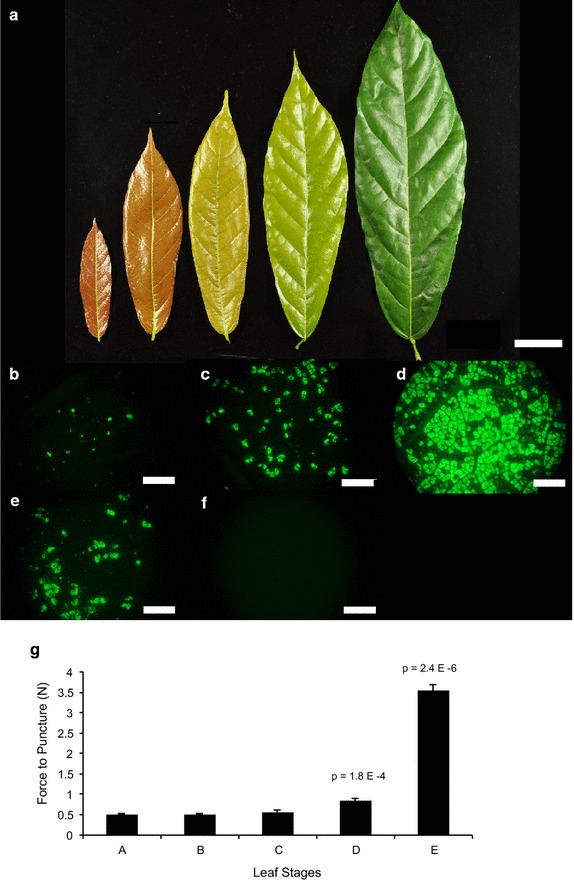
Leaf stages and force to puncture measurements. **a** Photograph displaying representative leaves of stages A (*leftmost*) to E (*rightmost*) collected from genotype Scavina 6. *Scale bar* represents 5 cm. **b**–**f** Representative photographs of EGFP fluorescence taken 48 h after infiltration of leaves (stages A–E) with *Agrobacterium*. *Scale bars* represent 1 mm. **g** Measurement of force to puncture for each leaf stage. *Bars* represent mean of five measurements, each representing one leaf from that stage. *Bars* represent standard deviation across five replicates. T test p values are shown above *bars* for Stage D and Stage E, which are comparisons of measurements of Stage C leaves with those of the older stages. Differences between Stage A and C and B and C were not significant

Cacao leaf stages were previously described [17]; however, as accurate determination of leaf stage is integral to successful transient transformation, we sought to more quantitatively describe the stages to enhance reproducibility of the protocol. In developing the protocol, we found that leaf age affected transformation efficiency, with both earlier and later developmental stages showing lower transformation success as measured by EGFP fluorescence. This resulted in our using Stage C leaves (Fig. 2a), which are expanded but still supple, for our transient transformation experiments. To demonstrate this observation, we transformed leaves of each stage, and 48 h after infiltration, photographed EGFP fluorescence (Fig. 2b–f). To measure leaf toughness, we used a force gauge and performed a punch test on leaves of stages A through E. Figure 2g shows the mean force to puncture, averaged across five leaves, for each leaf stage. Our protocols for collection and transformation and photographing of the five leaf stages, as well as the protocol for the force to puncture test, can be found in the Additional file 1. The data indicates that early in their development (through stage C), leaves do not significantly increase in rigidity. Stage D and E leaves, however, are measurably more rigid. Therefore, it is essential to take into account both leaf color (stage C leaves are bronze to light green) and rigidity to select leaves most likely to be successfully transformed.

**Fig. 3:**
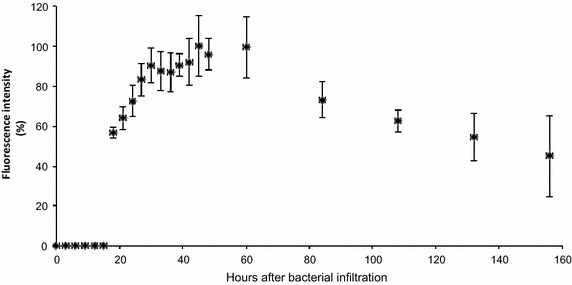
Time course of EGFP fluorescence intensity after infiltration of leaf tissue with *Agrobacterium*. Fluorescence is expressed as a percentage of the intensity measured at hour 45, the peak time point. *Error bars* represent standard deviation calculated from three biological replicates

In order to evaluate the rate at which cacao leaves infiltrated with *Agrobacterium* become transformed, we monitored expression of an EGFP transgene over a time course after infiltration. Leaves were imaged using a fluorescence stereo-microscope. Images were acquired immediately after transformation and every 3 h after bacterial infiltration (ABI) for the first 48 h, and at hours 60, 84, 108, 132, and 156. No EGFP fluorescence was detected until 18 h ABI. Fluorescence intensity increased until its peak at 45 h ABI, remained high until 60 h, and then steadily declined. EGFP fluorescence was quantified using ImageJ and is graphed as a percentage of the level detected at 45 h ABI (Fig. 3). Because the intensity peaks approximately 2 days ABI, this time point was selected to evaluate transformation success before proceeding into subsequent experiments. Further, our earliest detection of transient expression at hour 18 was consistent with findings in tobacco [24], and peak expression in our time course is consistent with results from transient transformation of *Arabidopsis* [25].

**Fig. 4:**
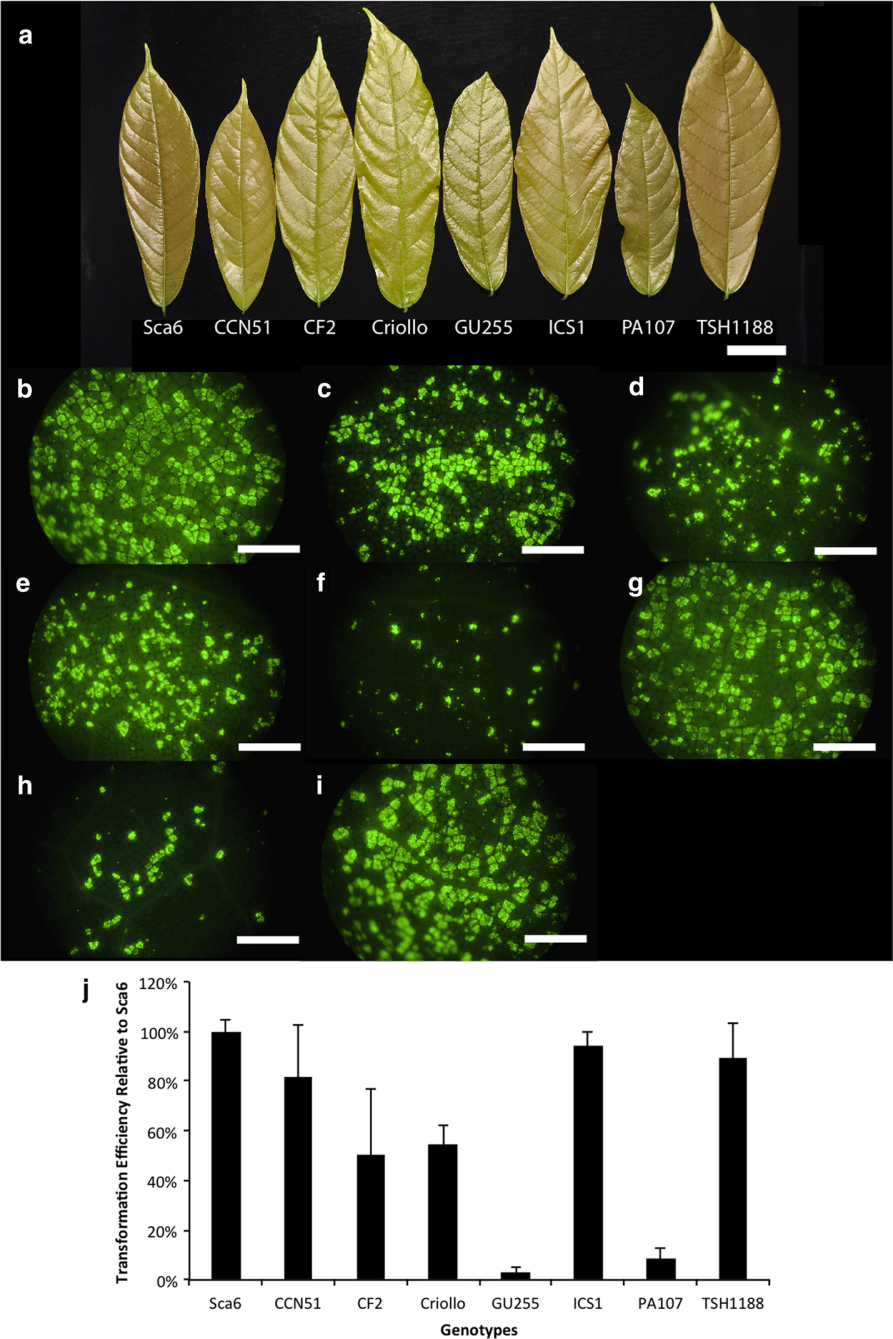
Transformation of eight cacao genotypes. **a** Photograph showing stage C leaves selected from eight cacao genotypes. Some genotype identifiers are abbreviated: *Sca6* Scavina 6, *Criollo* B97-61/B2, *ICS1* Imperial College Selection 1. *Scale bar* represents 5 mm. **b**–**h** Representative images of EGFP coverage 48 h after agrobacterium infiltration using the eight genotypes shown in panel **a**. *Scale bars* represent 1 mm. **b** Sca6; **c** CCN51; **d** CF2; **e** Criollo; **f** ICS1; **g** GU255; **h** PA107; **i** TSH1188. **j** *Bar* *graph* depicting transformation efficiency expressed as a percentage of that calculated for Scavina 6 samples. *Error bars* represent standard deviation calculated from three biological replicates

While the protocol was optimized for transformation of Stage C leaves [17] from genotype Scavina 6, it can be applied to other genotypes. Figure 4 includes photographs of stage C leaves from eight genotypes (Fig. 4a), as well as representative photographs showing transformation efficiency of these genotypes (Fig. 4b–i). In Fig. 4j, the transformation efficiency of each genotype was calculated and graphed relative to that measured in the Scavina 6 genotype. Our protocol for this genotype transformation optimization test, including calculation of transformation efficiency with ImageJ [26], can be found in the Additional file 1. While Scavina 6 exhibited the highest transformation efficiency, three other genotypes (CCN51, ICS1, TSH1188) had mean transformation efficiencies greater than 80 %, suggesting that our protocol could likely be easily applied to these varieties. Physiological differences between leaves of different genotypes may contribute to decreased efficiency, and some alterations to the protocol may be necessary to overcome low efficiencies of the transformation-recalcitrant varieties. We have also previously noted that Scavina 6 leaves appear to remain green and survive longer in petri dishes than other genotypes [13], so it may be generally more suitable to long-duration experiments.

After identifying successfully transformed leaves, subsequent experiments including RNA extractions, pathogen inoculations, and lipid extractions can be performed, as have been described [13, 14, 19, 22]. Leaves will show significant desiccation 5–7 days after being detached from plants; therefore, experiments should not require more than 3–5 days after transformation success is confirmed. Other than this limitation, the transformation strategy can be widely applied to gene characterization studies. In addition to the transformation protocol, we also provide here a detailed methodology for infection of leaves with pathogen after transformation. In addition to the transformation protocol, we also provide here a detailed methodology for infection of leaves with pathogen after transformation.

**Fig. 5:**
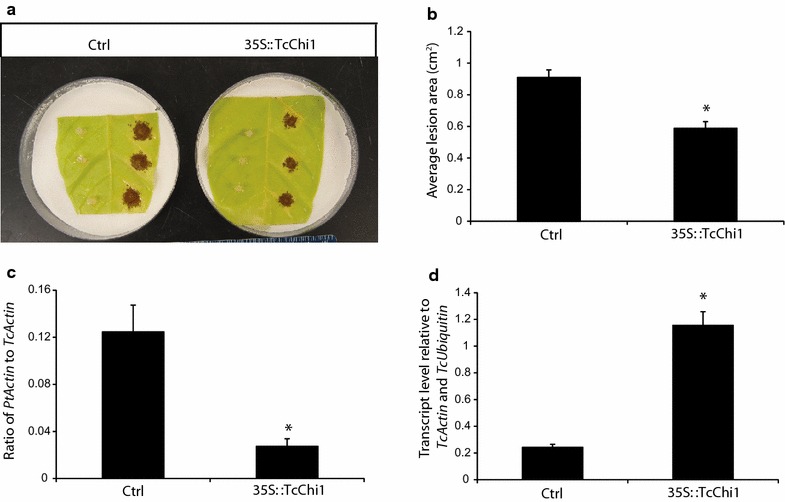
Functional analysis of *TcChi1*. **a** Representative images of lesions from control (Ctrl, transformed with pGH00.0126) and leaves transiently transformed to overexpress *TcChi1* 2 days after *Phytophthora tropicalis* inoculation. *Scale bar* represents 1 cm. **b** Average lesion areas from control and *TcChi1* overexpressing leaves were measured 3 days after inoculation using ImageJ. *Bar charts* represent the mean ± SE of measurements from 12 lesion spots from four leaf discs of each genotype. **c** Pathogen biomass was measured at the lesion sites by qPCR to determine the ratio of pathogen DNA to cacao DNA 2 days after inoculation. *Bar charts* represent four biological replicates, each with three technical replicates. **d** qRT-PCR analysis of *TcChi1* transcript 2 days after vacuum infiltration. Data represent mean ± SE of three biological replicates. The *asterisks* denote a significant difference determined by single factor ANOVA analysis (p < 0.05)

In Fig. 5, we have included additional data demonstrating the effect of transient overexpression of a previously described cacao chitinase gene [15]. Our protocol for these experiments is available in the Additional file 1. Two constructs were used for the transient transformation, pGH00.0126 (GenBank: KF018690), in which EGFP is driven by the CaMV 35S promoter, and another (pGAM00.0511, described in [15]) which has an additional cassette containing a cacao chitinase gene (Tc02_g003890) under the CaMV 35S promoter. Chitinase overexpression using this system resulted in decreased lesion size after infection with *Phytophthora tropicalis* (Fig. 5a, b), a decrease in the ratio of pathogen to cacao DNA detected in the tissue (Fig. 5c), and an approximately six-fold increase in chitinase transcript abundance as assessed by qRT-PCR (Fig. 5d).

We have previously documented differences in basal response to infection to the pathogen *Phytophthora tropicale* in leaf tissue taken from different cacao genotypes using our detached leaf infection assay [13]. While the earlier analysis focused on only one model tolerant genotype (Scavina 6) and model susceptible genotype (ICS1), here we present preliminary data expanding this analysis to 17 genotypes. Additional file 1: Fig. S1 shows box and whisker plots representing the area of infected tissue 72 h after inoculation using the detached leaf infection protocol described here. Additional file 1: Fig. S2 contains representative photographs of the infected leaf tissue from the 17 genotypes. The dramatic differences in susceptibility highlight the need for application of the transient transformation protocol to a wide range of genotypes in order to understand better the genetics underlying differential defense response.

## Reagents and equipment

For transformation:

- *Agrobacterium* is cultured in 523 media, and induced as previously described [27]. Recipes for these media can be found in Table 1.

**Table 1:** Media recipes for *Agrobacterium* growth and induction and pathogen growth

Reagent	Amount per liter
523 Medium (1 L)^a^	
Sucrose	10 g
Casein enzymatic hydrolysate	8 g
Yeast extract	4 g
K_2_HPO_4_ anhydrous	2 g
MgSO_4_ anhydrous	0.15 g
Induction medium (recipe per 30 mL volume)^b^	
Liquid ED (recipe described in [30])	30 mL
0.1 M acetosyringone (Sigma Cat. # D134406)	30 μL
l-Proline	0.00465 g
20 % V8 Media (1 L)^c^	
Bacto Agar	15 g
Calcium carbonate (CaCO_3_)	3 g
Campbell’s V8 Vegetable Juice	200 mL

^a^Add distilled water to 1 L. Adjust pH to 7.1 and autoclave

^b^Adjust pH to 5.25–5.3 using 0.1 M KOH. Discard if pH exceeds 5.32. Do not adjust pH using HCl. Prepare induction medium on morning of leaf infiltration experiment. Use liquid ED less than 30 days old

^c^Add distilled water to 1 L. Adjust pH to 7.1 and autoclave. Shake frequently while pouring into petri dishes to maintain homogeneity of media. Pour about 20 mL of media into each plate to ensure that agar plugs and do not fall during leaf assay
- A Fast PES Filter unit (Thermo Scientific, Cat. No. 124-0045) is used to sterilize induction media.
- Before infiltration of leaves, Silwet L-77 (Lehle Seeds, Cat. No. VIS-01) is added to *Agrobacterium* cultures to act as a surfactant.
- Plants used for these experiments are greenhouse-grown on The Pennsylvania State University, University Park campus under previously described growth conditions [28]. They are also described in the Additional file 1.
- After leaves are infiltrated with *Agrobacterium*, they are maintained in a controlled environment at 25 °C with 50 % relative humidity and a 12 h/12 h light dark cycle. Light levels are maintained at 55 µmol m^−2^ s^−1^, using fluorescent bulbs 4100 K Kelvin ratings. Higher light levels did not affect transgene expression, but did lead to faster desiccation of leaves.
- Gast G582DX Vacuum Pump.
- Science-Ware vacuum desiccator (Cat# 420270000).
- Whatman grade 5 qualitative filter paper, 90 mm diameter discs (Cat# 1005-090).
- Sterile 100 mm × 20 mm petri dishes (Fisher Brand Cat# FB0875711Z).
- Paraplast Plus tissue embedding medium (McCormick Scientific Cat# 39503002).
- Orbital shaker.
- General lab supplies: pipettors, pipette tips, Parafilm, paper towels.

For pathogen bioassay:

- Pathogen subcultures (age depends on pathogen).
- Appropriate media for pathogen growth (recipe for 20 % V8 media is listed in Table 1).
- Laminar flow hood.
- Atomizer of sterile water.
- 3 mm diameter cork borer, 6 mm diameter cork borer, 1.5 cm diameter cork borer.
- General lab supplies: forceps, probe, petri dishes.

## Protocol

### Preparation of *Agrobacterium* working stocks for transformation

*Timing* Approximately 1 h, plus overnight incubation

1. Prepare 523 media (see “Reagents and equipment” section).
2. *Agrobacterium* for transformations are cultured using working stocks at OD600 to ensure that cultures grow at consistent rates. To create working stocks, inoculate freezer stocks of AGL1 colony containing desired plasmid in 2 mL 523 medium with appropriate antibiotic and shake overnight at 200 rpm, 25 °C.
3. Measure OD at 600 nm. Let the culture grow until OD600 is 1, or dilute to 1 with 523 media if above. Take 750 µL culture and transfer into a sterile 1.5 mL tube. Add 250 µL of 60 % glycerol. Mix well. Aliquot 100 µL of the mixture into cyrovial tubes. Store at −80 °C.

### Day 1: inoculation and incubation of *Agrobacterium* culture

*Timing* Approximately 10 min, plus overnight incubation

1. Thaw a 100 µL working stock of AGL1 for each desired plasmid.
2. Inoculate 90 µl of AGL1 stock into 30 ml of 523 media with Kanamycin (50 mg/ml) in a sterile 125 ml Erlenmeyer flask covered with aluminum foil.
3. Shake in the dark overnight at 200 rpm at 25 °C (approximately 16 h).

### Day 2, part I: virulence induction of *Agrobacterium culture*

*Timing* Approximately 1 h of active time, plus 5 h incubation

1. For every 30 mL culture of Agrobacterium, prepare 30 mL of induction media (see “Reagents and equipment” section). Vacuum sterilize the induction media using Fast PES Filter unit (Thermo Scientific, Cat. # 124-0045).
2. Measure OD of overnight cultures at 600 nm. Use 523 media as a blank. Wait for all cultures to reach OD of 1. Remove those that have passed this point from the shaker to prevent overgrowth. If OD has passed 1.3, discard cultures. If OD is between 1 and 1.3, dilute to OD 1.0 with 523 media.
3. Transfer the entire culture to a 50 mL centrifuge tube. Centrifuge the *Agrobacterium* at 1500×*g*, 25 °C, for 17 min to pellet the bacteria.
4. Discard supernatant, gently pipette and vortex to re-suspend cultures using 30 mL of induction media and transfer to new 250 mL flasks. Ensure that pelleted bacteria are thoroughly suspended in the solution.
5. Shake at 100 rpm at 25 °C for 5 h in darkness. During this step, collect leaves and prepare plates.

### Day 2, part II: plate preparation and leaf selection

*Timing* Approximately 1 h

*Note* Plate preparation and leaf collection will take approximately an hour, so perform these steps about 4 h after beginning *Agrobacterium* induction, typically early in the afternoon.

**Fig. 6:**
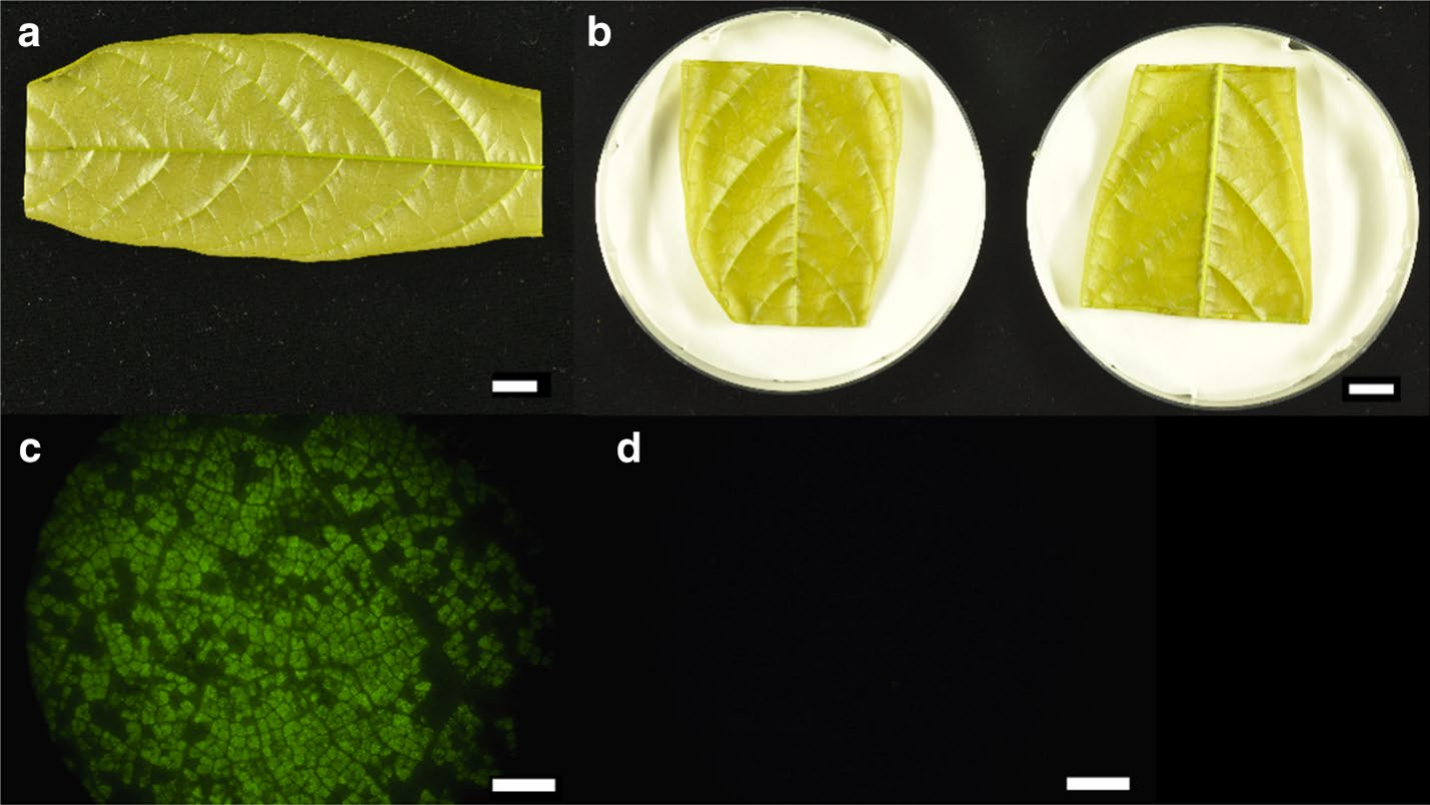
Images representing stages in leaf transformation process. **a** Stage C cacao leaf with tip and base removed. *Scale bar* represents 1 cm. **b** Two halves of a cacao leaf placed into petri dishes with wet paper towel and filter paper. *Scale bar* represents 1 cm. **c** Ideal EGFP coverage seen 48 h after vacuum infiltration of leaves with *Agrobacterium*. *Scale bar* represents 1 mm. **d** Photograph of untransformed leaf tissue using GFP filter. *Scale bar* represents 1 cm

1. Place ten Paraplast Plus chips onto a glass petri dish and apply low heat (~56 °C) until they melt.
2. For each plate, fold a paper towel into a square, and cut off the corners to fit it into a 100 × 20 mm petri dish. Place Whatman #5 filter paper on top of the paper towel and gently press down to create a flat surface. Add 10 ml of sterile water to the plate to maintain humidity.
3. Collect Stage C leaves from greenhouse grown plants. It is essential to the success of the experiment that leaves are soft and supple, and Stage C leaves are bronze to light green in color. Cut the petiole to remove the leaf from the plant without damaging the leaf’s surface area. Place the leaves in a sealable plastic bag containing wet paper towels to maintain humidity.
4. Cut leaves with a scalpel to produce leaf two sections. First, the tip and base of the leaf are removed (Fig. 6a). Next the leaf is divided into two sections of equal size. Ensure that each section is large enough to accommodate subsequent experiments (i.e. inoculation with pathogens). As leaves are cut, seal the cut edges by dipping into melted paraffin. This will limit desiccation from exposed veins. Place the leaf discs onto plates for temporary storage, abaxial side up (Fig. 6b) and close the plates. Let sit on the lab bench until induction of *Agrobacterium* is complete.

### Day 2, part III: vacuum infiltration

*Timing* Approximately 1–2 h, depending on replicate number

1. After 5 h in the incubator, add pure Silwet L-77 to the *Agrobacterium* culture to a final concentration of 0.02 % (for a 30 ml culture, this is 6 µL of Silwet). Silwet L-77 is necessary for successful transformation. Our preliminary results indicated that higher Silwet L-77 concentrations do not increase transformation success rates.
2. Pour induced *Agrobacterium* suspension onto 100 mm × 20 mm petri dishes labeled with the construct name on the bottom of the plate as lids are removed during infiltration.
3. With lids removed, place the petri dishes of induced *Agrobacterium* into the desiccator.
4. Select a leaf section to be placed into each dish of *Agrobacterium*, abaxial side down. The other section of the same leaf should receive the other treatment. *Agrobacterium* containing control and experimental vectors are typically infiltrated into their respective leaf sections concurrently. Place the lid on the desiccator.
5. Vacuum-infiltrate the leaves.
  - Turn the stopcock valve to open airflow between vacuum pump and desiccator. Start a timer as pressure begins to build.
  - Ensure that the pressure reaches −22 in. Hg on pressure gauge. Wait 10 min. As leaves sit in vacuum, small air bubbles should appear at the edges of the leaf.
  - Turn the stopcock valve to release vacuum inside desiccator.
6. Using separate tweezers and paper towels for each construct, gently remove the leaf disc from the desiccator, blot dry in paper towels, and hold up to light to look for flooding of cells to assess infiltration success.
  - Spots of translucence on the underside of the leaf indicate successful infiltration, which correlates with high EGFP and transgene expression on day 4.
  - If none of the leaves have noticeable flooding, the experiment transformation will likely be unsuccessful.
7. Place the leaf abaxial side up onto its petri dish from step 2. Ensure complete contact between the leaf and the filter paper by placing one corner down first and slowly lowering the leaf so that it adheres to the filter paper. Place lid on the petri dish, and seal it with parafilm.
8. Repeat steps 2–6 for all remaining leaf discs.
9. Incubate leaves in a growth chamber at 25 °C with 12 h:12 h light dark cycle for 2 days with a light intensity of 55 µmol m^−2^ s^−1^. Higher light levels were found to lead to faster deterioration of leaf tissue.

### Day 4: evaluating EGFP expression

*Timing* Approximately 1 h

1. Forty-eight hours after infiltration, gather leaf tissue.
2. Using a fluorescence microscope, scan the surface area of each leaf for EGFP as previously described [13]. In order for leaves to be useful for subsequent experiments, at least 80 % of the surface area of the leaf should fluoresce, and there should be no large patches of tissue not expressing EGFP. Representative image of EGFP fluorescence over a small area of leaf tissue is included in Fig. 6c. Coverage across the entire surface of the leaf should match this level of expression. Background fluorescence of cacao leaf tissue is minimal (6D), making transformed regions readily identifiable.
3. Leaf-to-leaf physiological variability may contribute to some variability in downstream experiments. Consequently, only pairs of leaf sections that both pass the EGFP threshold should be retained. Any pairs of leaves where either has less than 80 % of its surface showing EGFP can be discarded.

### ***Phytophthora*** bioassay

#### **Part I: subculturing pathogen**

*Timing* Approximately 15 min

1. Sterilize a laminar flow hood with UV light for 2 min, and wipe the area with 70 % ethanol.
2. Sterilize the 6 mm diameter cork borer and forceps using 70 % ethanol and flame. Let cool briefly.
3. Use the cork borer to create agar plugs in a mature plate of pathogen (Fig. 7a, b).

**Fig. 7:**
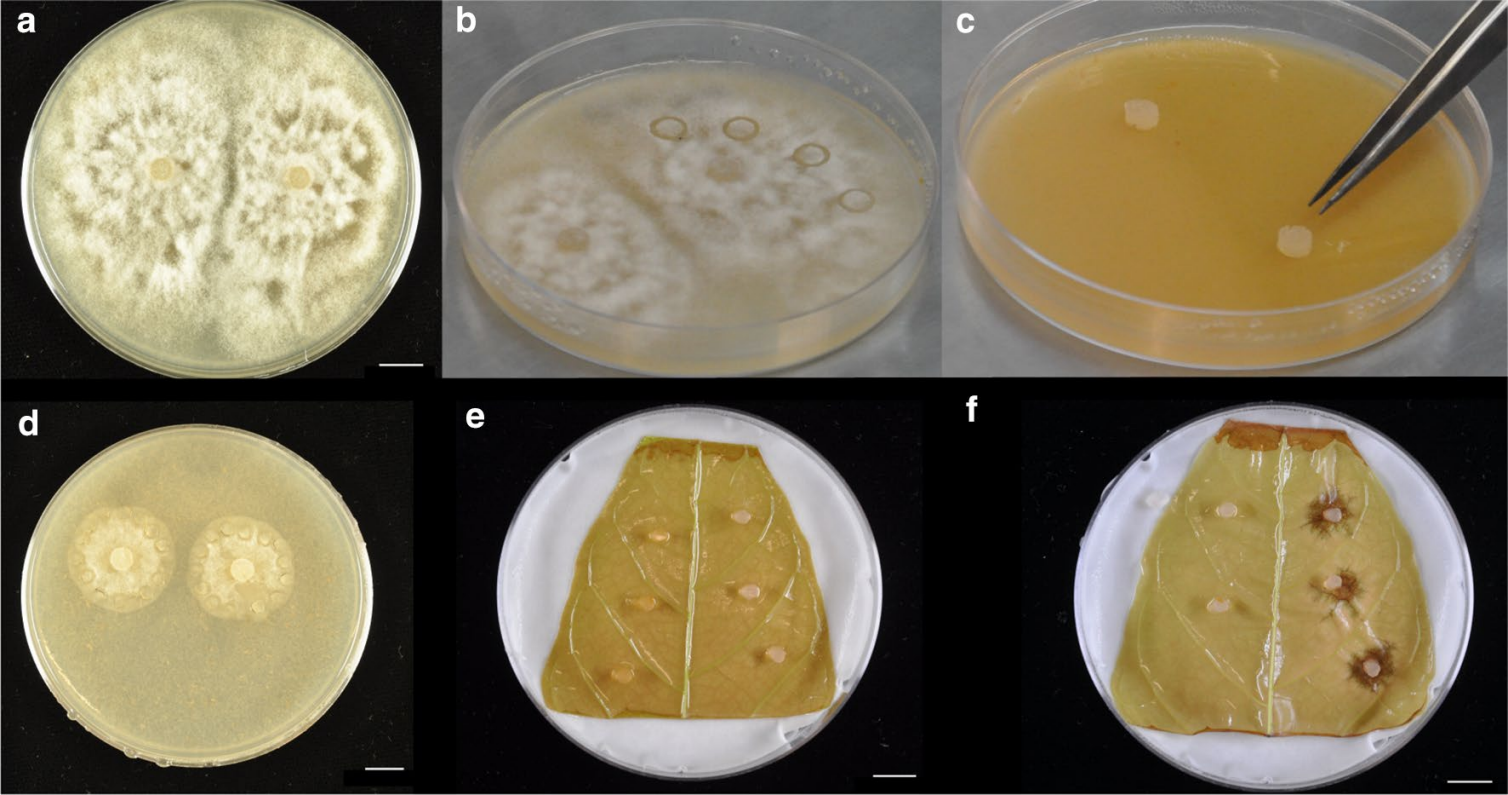
Images representing leaf infection process. **a** Mature (approximately 1 week since inoculation) plate of the cacao pathogen *Phytophthora palmivora*. **b** Plate of *P*. *palmivora* with four agar plugs bored into V8 media. **c** Inoculation of a new plate of 20 % V8 media by transferring agar plugs, pathogen side down, onto the media. **d** Typical size of pathogen growth 48 h after inoculation of new plate. Agar plugs are bored around the edges of the cultures to be used for leaf inoculation. **e** Inoculation of a Stage C cacao leaf with pathogen. Control (media only) plugs are placed on the *left side*, plugs containing pathogen are placed on the *right*. **f** Lesion development 48 h after inoculation. All *scale bars* represent 1 cm
4. Transfer agar plugs, pathogen side down, to a new plate of V8 media (Fig. 7c).
5. Incubate the leaves at 28 °C, 12:12 light/dark cycle for 48 h.

#### **Part II: inoculation of leaf tissue**

*Timing* Approximately 1 h

1. Sterilize the laminar flow hood with UV light for 2 min, and wipe the area with 70 % ethanol.
2. Sterilize the 3 mm cork borer with ethanol and flame. Let it cool, and then use it to bore holes into a plate of V8 media with no pathogen. These agar plugs will be used to demonstrate that placing the media on the leaves does not result in formation of a lesion.
3. Re-sterilize the cork borer and let cool briefly. Use it to bore agar plugs around the outside edges of the pathogen culture, as shown in Fig. 7d. Creating plugs from the edges of the culture ensures that the pathogen is actively growing, and that all agar plugs used will be equally virulent.
4. Place agar plugs on the leaf as shown in Fig. 7e. First, sterilize forceps and a probe, and let them cool. Use it to place three V8 agar plugs without pathogen along the left side of the leaf in a line parallel to the midvein. Place agar plugs with containing pathogen mycelia along the right side of the leaf. Ensure that the pathogen’s side of the agar plug is in contact with the leaf. Avoid placing an agar plug near the primary or secondary veins as they affect the shape of lesion growth, or too close to another plug so that lesions do not coalesce. For reference, when harvesting lesions, a 1.5 cm diameter disc will be cut for each lesion.
5. Repeat the inoculation for all remaining leaf sections
6. Use the atomizer to spray each leaf with sterilized water. Ensure that the leaf was uniformly misted.
7. Seal plates with parafilm, handling carefully so as to not disturb agar plugs.
8. Incubate leaves in a growth chamber at 25 °C with 12 h:12 h light dark cycle for 2 days with a light intensity of 55 µmol m^−2^ s^−1^.

#### **Part III: leaf photography and tissue collection**

*Timing* Approximately 2 h

1. If inoculation was successful lesions will have developed after 48 h (Fig. 7f). Photograph the leaves, including a ruler as reference for measurement. Use ImageJ [26] to trace the area of the lesions, and average the three lesions on a leaf to serve as a biological replicate.
2. Remove agar plugs using forceps. Follow appropriate guidelines for disposal of the pathogen.
3. Cut lesions of each leaf using a 1.5 cm diameter cork borer with location of agar as center. Using a sharpened cork borer will prevent leaf tearing. For each leaf, place the three leaf discs into a 2 mL microfuge tube. Flash freeze the tissue with liquid nitrogen, and store at −80 °C. This tissue will be used for DNA extractions and subsequent qPCR to compare relative abundance of pathogen to host DNA within the infected tissue.
4. Use a scalpel to excise the “donut” of tissue around where the lesions developed. Again, place this tissue in a 2 mL microfuge tube, flash freeze, and store at −80 °C. This tissue can be used for RNA extraction as previously described [19] to verify overexpression of the transgene, and to compare expression level of other genes of interest between the transgene-overexpressing samples and those treated with vector control.

## Conclusions

The transient transformation procedure described here offers a rapid means of performing functional genetic characterization studies on cacao, a long generation tree crop of significant economic importance. The strategy has already been applied to several studies [13, 14, 19, 20, 22], which were studies investigating single gene overexpression and knockdown. The cacao transient transformation protocol was first described by Shi et al. [22]. In this study, the transcription factor NPR3 was shown to be a negative regulator of the cacao defense response by using transient microRNA-mediated knockdown of the TcNPR3 transcript in cacao leaves followed by *Phytophthora* inoculation assays. The protocol was also applied to the study of cacao defense response by Mejia et al., who demonstrated that overexpression of a cacao gene induced by presence of the endophyte *Colletotrichum tropicale*, decreased susceptibility to *Phytophthora* infection [20]. This result suggested that the presence of endophytes in cacao leaves confers a mutualistic enhanced defense response to attack by pathogens [20]. The transient transformation was also applied by Fister et al. in a study demonstrating the positive role of NPR1, the master regulator of systemic acquired resistance, in cacao’s response to infection by *Phytophthora* [13]. Helliwell et al. applied cacao leaf transient transformation to show that expression of phosphatidylinositol-3-phosphate binding proteins can decrease susceptibility to infection by competitively inhibiting pathogens’ effector proteins’ abilities to bind to host cell membranes [14]. Finally, Zhang et al. used the transient transformation strategy to characterize the role of the transcription factor TcLEC2, transiently overexpressing it in leaves to demonstrate its role in regulating genes related to embryo development [19]. While the previous work focused on transformation of only the Scavina 6 genotype, we demonstrate here that the protocol can be applied to transform other genotypes. A recent study reported that the genotypes CCN51 and TSH1188 were used as parents in a mapping population to identify resistance genes for witches’ broom disease [29]. Here we demonstrate the ability to transiently transform both of these genotypes (among others), with high efficiency, which would allow screening of defense gene overexpression in the genotypes of interest. Using the infection assay described here, we have already demonstrated variable defense responses between genotypes [13], and here we provide preliminary data on a wider array of cacao genotypes. These data reflect the wide range in susceptibilities different genotypes can exhibit. Application of our transient transformation protocol will enable future work to probe the genetic mechanisms underlying these differences. Further, the development and application of this leaf transformation study enables these types of gene characterization studies to be performed rapidly and at lower cost than through the creation of stably transgenic plants. Without this strategy for rapid gene testing, similar analyses require several years and extensive resources in order to generate stably transgenic cacao trees. The transient transformation strategy is also in the process of being adopted for altering expression of multiple genes by including additional cassettes, and will also be used to develop CRISPR/CAS9-mediated genome editing in cacao leaves.

## Additional file

10.1186/s13007-016-0119-5 Multiple genotype infection graph – Box and whisker plots displaying lesions sizes 72 hours after inoculation of stage C cacao leaves of 17 genotypes with Phytophthora tropicale mycelia. **Fig. S2.** Photographs of infected leaf tissue of diverse genotypes – Representative photographs 72 hours after inoculation of leaf tissue with Phytophthora tropicale. Scale bars represent 1 cm. Supplemental Methods – Descriptions of protocols used for plant growth, transformation and photography of the five leaf stages, the force to puncture test, transformation of the eight cacao genotypes, and evaluation of effects of TcChi1 overexpression.
