# Supplementary material for: Protocol: transient expression system for functional genomics in the tropical tree Theobroma cacao L
Source: Plant Methods. 2016 Mar 11;12:19. doi: 10.1186/s13007-016-0119-5 (PMC4788949; doi:10.1186/s13007-016-0119-5)
Supplement: Supplementary file 1 — 10.1186/s13007-016-0119-5 Multiple genotype infection graph – Box and whisker plots displaying lesions sizes 72 hours after inoculation of stage C cacao leaves of 17 genotypes with Phytophthora tropicale mycelia. Fig. S2. Photographs of infected leaf tissue of diverse genotypes – Representative photographs 72 hours after inoculation of leaf tissue with Phytophthora tropicale. Scale bars represent 1 cm. Supplemental Methods – Descriptions of protocols used for plant growth, transformation and photography of the five leaf stages, the force to puncture test, transformation of the eight cacao genotypes, and evaluation of effects of TcChi1 overexpression. [file 13007_2016_119_MOESM1_ESM.pdf]

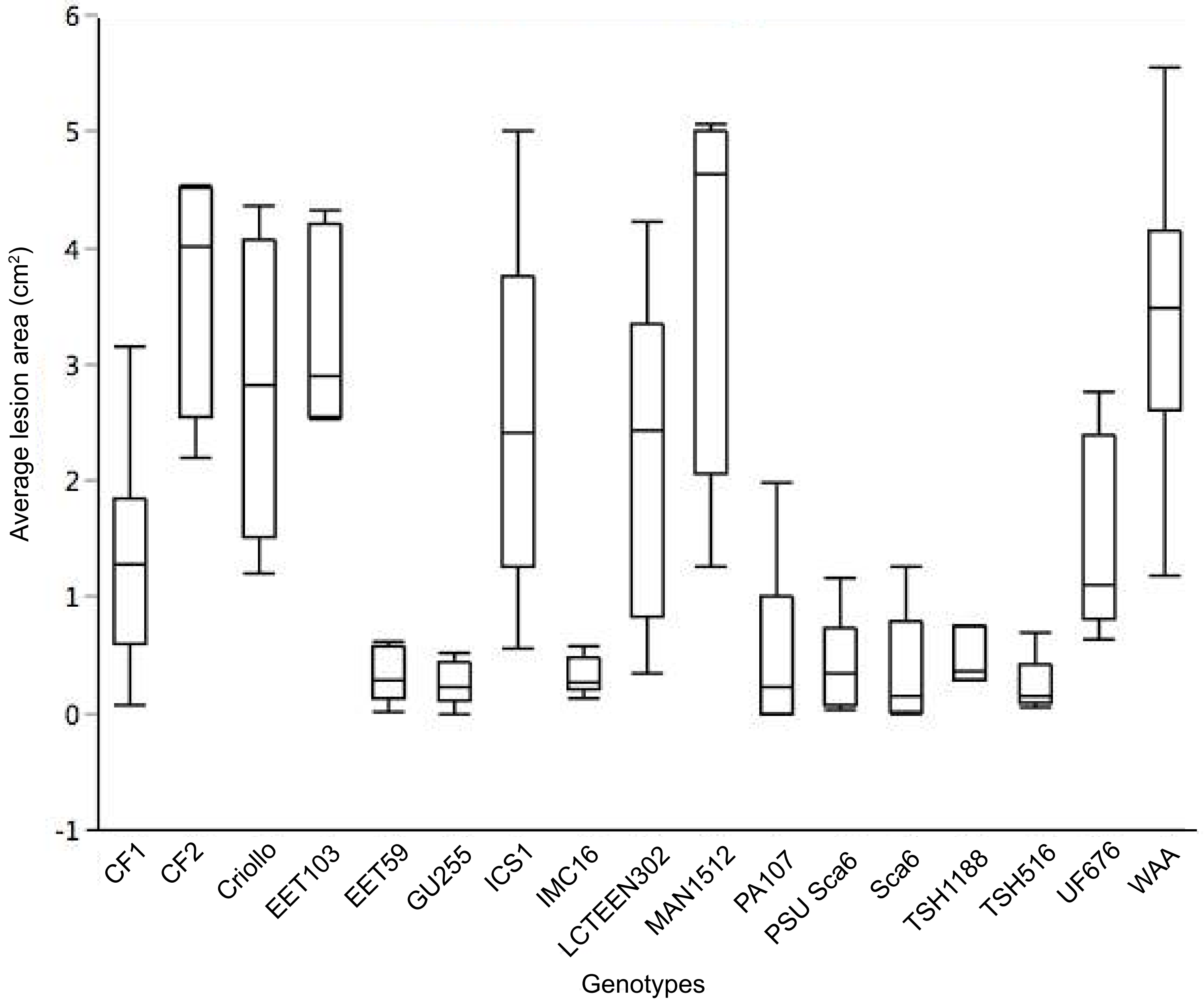

Supplemental Fig. S1 - Box and whisker plots displaying lesions sizes 72 hours after inoculation of stage C cacao leaves of 17 genotypes with *Phytophthora tropicalis* mycelial plugs. Number of replicates per genotype are as follows: CF1: 9, CF2: 4, Criollo:4, EET103: 7, EET59: 8, GU255: 5, ICS1: 9, IMC16: 8, LCTEEN302: 8, MAN1512: 4, PA107: 10, PSU Sca6: 11, Sca6: 9, TSH1188: 3, TSH565: 5, UF676: 5, WAA: 8.

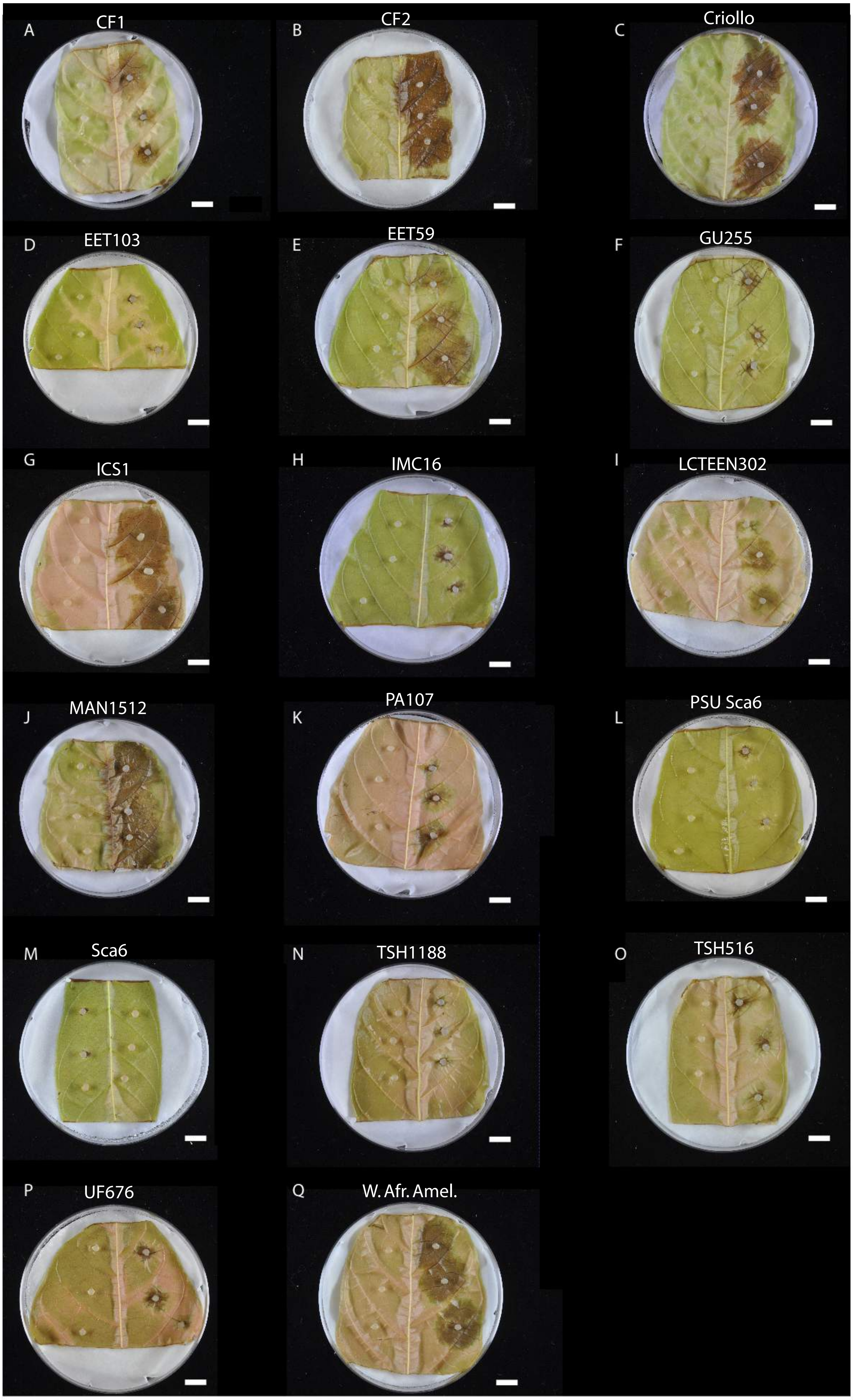

Supplemental Fig. S2 – Representative photographs 72 hours after inoculation of leaf tissue with *Phytophthora tropicalis*. Scale bars represent 1 cm.

Supplemental Methods

Plant and their growth conditions

Scavina 6 leaves used for leaf stage photographs, force to puncture tests, chitinase overexpression, and the transformation and infections depicted in Figures 6 and 7 were collected from young (< 5 year old) greenhouse grown plants generated by rooted cutting. The leaves from all genotypes shown in Figure 4 were taken from mid-aged (~10 year old) greenhouse-grown trees generated by rooted cutting. Growth conditions were previously described [28]. Briefly, plants are grown in a silica and sand mix for adequate drainage and kept at 60% humidity. They are kept at a 12 hr / 12 hr day/night cycle with days at 29°C and nights at 26°C. They are irrigated with one-tenth-strength Hoagland's nutrient solution to promote growth.

Transformation of Leaf Stages A-E

Three leaves of each leaf stage were collected from greenhouse-grown Scavina 6 trees. One leaf of each stage was included in the photograph in 2A, which was taken using a Nikon D90 camera using a 1/30 second exposure time, aperture of f = 5.6 as described [13]. Then the leaves were cut and placed in petri dishes, and the transformation was carried out as described in the protocol. Forty eight hours after *Agrobacterium* infiltration, EGFP fluorescence of the leaves was observed. One photograph was taken to represent the average EGFP coverage seen across the three biological replicates. Previously described microscope and camera settings were used [16].

Force to puncture test of leaf toughness

Five leaves of each stage were collected from greenhouse-grown Scavina 6 trees. The leaves were clamped so that they were held tautly and the probe of a Dillon Model GS force gauge (Dillon, Fairmont, MN) could be lowered through the tissue. In taking the measurements, we avoided the midvein and secondary veins in order to obtain uniform measurements. Each leaf was penetrated six times, and the average of those measurements was used as a biological replicate. Therefore, the average forces to puncture shown in Figure 2G represent five biological replicates.

Transient transformation of eight cacao genotypes

Three stage C leaves of eight genotypes were collected from greenhouse-grown trees. One leaf from each genotype was photographed (Fig. 4A) to depict the morphology of the leaves. The photograph was taken using a Nikon D90 camera using a 1/30 second exposure time, aperture of f = 5.6 as described [13]. The leaves were subsequently cut and the transformation was carried out as described in the protocol. Forty-eight hours after *Agrobacterium* infiltration, leaves were screened for EGFP fluorescence and photographed as described [16]. Each leaf was photographed once, and an area of tissue was selected that represented transformation success across the surface of the leaf. In order to compare transformation success between genotypes, we analyzed the images using ImageJ [26]. For each photograph, the EGFP fluorescing area was selected by using the threshold function to select all pixels with brightness values between 105 and 255. The measured area for each sample and the

standard deviation for the genotype were divided by the average of the three values from the Scavina 6 samples in order to express transformation success as relative to that of Scavina 6.

Functional analysis of transient *TcChi1* overexpression

In order to test the effect of transient overexpression of a cacao chitinase gene (Tc02_g003890) [15], the transient transformation protocol was applied. The effect of overexpression was measured as previously described [13]. Briefly, 48 hours after *Agrobacterium* infiltration, leaves were screened for EGFP expression. Successfully transformed (>80% EGFP coverage) leaf tissue was collected for RNA extractions. Another set of leaves with >80% coverage were inoculated using mycelial plugs of *Phytophthora tropicalis.* After 72 hours, photographs were taken (Fig. 5A) in order to measure lesion size with ImageJ Fig 5B). Lesions were collected and DNA and RNA extractions were performed as described [13]. The collected RNA was used for cDNA synthesis and qRT-PCR to verify transgene overexpression. Expression of *TcChi1* (Tc02_g003890) was calculated relative to *TcActin* and *TcUbiquitin* using the Taqman ABI 7300 Sequence Detection System (Applied Biosystems Inc, Foster City, CA, USA), using the following primers: *TcChi1:* 5′ — GCTCGTGGCTTCTATACCTATG, 3′ —AGCAGCAACTTCCCTCTTG, Probe — TCTTTCCCTGCCTTCGCTACAACC; *TcActin*: 5′—GATTCAGATGCCCAGAAGT CTTG, 3′—TCTCGTGGATTCCAGCAGCT, Probe—CCAGCCC TCGTTGTGGGAAAGG; *TcUbiquitin*: 5′ —AGGCCTCAACTG GTTGCTGT, 3′—ACCGGCAAGACCATCACTCT, Probe— CGAGAGCAGCGACACCCATCGACA. The extracted DNA was used in qPCR reactions to quantify the relative amount of pathogen and plant DNA in the tissue. Again the ABI 7300 was used, along with the specific primers for *P. tropicalis Actin* (F: GACAACGGCTCCGGTATGTGCAAGG and R: GTCAGCACACCACGCTTGGACTG) and cacao *Actin* (F: AGGTGGAGATCATTGAAGGAGGGT and R: ACCAGCGGTCATCACAAGTCACAA).
